# Examining the role of social workers within clinical metabolic genetics care

**DOI:** 10.1016/j.ymgmr.2026.101326

**Published:** 2026-06-08

**Authors:** Soo Shim, Diana Cory, Gailon Wixson, Abigail Hata, Allison Werner-Lin

**Affiliations:** a Ann & Robert H. Lurie Children's Hospital, Chicago, IL, USA; b Columbia University, New York, NY, USA; c Seattle Children's Hospital, Seattle, Washington, USA; d Amgen, Thousand Oaks, California, USA; e School of Social Policy and Practice, University of Pennsylvania, Philadelphia, PA, USA

**Keywords:** Inborn errors of metabolism, Metabolic clinic, Genetics, Multidisciplinary care, Social worker, Social determinants of health

## Abstract

Optimal care for individuals with inborn errors of metabolism (IEMs) requires a well-integrated multidisciplinary care team in which each member contributes specialized expertise to address the complex medical, psychosocial, and logistical challenges faced by patients and their families and caregivers. Among these team members, metabolic social workers offer essential competencies that position them as integral to patient care, with the potential to improve care and outcomes for patients with IEMs. Social workers are uniquely equipped to identify and address social determinants of health, advocate for accommodations in daily living and educational settings, and provide psychosocial support for patients and families during critical times. Their integration into models for chronic conditions relevant to IEMs has been associated with benefits such as improved treatment maintenance, enhanced quality of life, and reduced health care utilization. Despite real-world evidence highlighting the benefit of social workers in rare and complex diseases, including IEMs, many practices struggle to secure dedicated social work support. Here, we aim to underscore the importance of integrating social workers into the metabolic team by outlining the breadth of their responsibilities and highlighting how their specialized skill set and experience help deliver comprehensive care for patients with IEMs.

## 1 Introduction

Inborn errors of metabolism (IEMs) comprise a heterogenous group of genetically inherited disorders that disrupt essential metabolic pathways and are broadly categorized into intoxication disorders, energy deficiency disorders, and disorders involving complex molecule metabolism, each with distinct pathophysiological mechanisms and clinical manifestations [1], [2], [3]. Although individual IEMs are rare, they are collectively common and affect approximately 1 in 2500 live births [1]. Patients can present at any age, with heterogenous symptoms ranging from mild to severe, including failure to thrive, cardiac failure, cerebral edema, coma, and death [1], [3], [4]. Because clinical presentation varies and can overlap with many other diseases, IEM diagnoses can be missed. As a result, the mortality rate for patients with IEMs is generally considered high, especially for untreated or undiagnosed cases [5].

IEMs affect patients and families across medical, emotional, and social dimensions. Given the diagnostic complexity, lifelong care needs, and psychosocial impact of IEMs, optimal management requires a multidisciplinary team. The multidisciplinary metabolic team is typically composed of medical and laboratory biochemical geneticists, specialized physicians and advanced practice nurses, metabolic dietitians, and genetic counselors. Social workers are also integral members of the care team, helping families navigate complex systems, manage psychosocial stressors, and access necessary support, particularly as patients face lifelong challenges that extend well beyond medical care [6]. While many consider social workers an essential component of an ideal multidisciplinary metabolic care team, only one-third of metabolic clinics have a dedicated social worker [7]. Metabolic social workers support key aspects of comprehensive, patient-centered care, including minimizing barriers to care, providing education and counseling throughout the patient journey, identifying and addressing social determinants of health (SDOH), and offering support for mental health challenges (Table 1) [6].

**Table 1:** Roles and potential impacts of a metabolic social worker.

	Examples of Care Needs	Social Worker Role	Potential Impact
**Diagnosis**	Provide emotional support during diagnosis disclosure	Normalize emotional responses; offer coping strategies	Helps patients and caregivers process shock and grief; builds trust and engagement
	Conduct psychosocial assessment of the family system	Identify strengths, stressors, caregiving capacity, and unmet needs	Informs individualized care and detects early psychosocial risks
	Facilitate immediate resource navigation	Assist with insurance enrollment, WIC, metabolic formula, and medical supplies	Reduces delays in treatment initiation and lowers patient and caregiver stress
	Screen for social determinants of health	Evaluate needs related to housing, transportation, employment, and food access	Identifies structural barriers to sustaining treatment and clinic attendance
	Offer crisis counseling and grief support	Provide emotional stabilization during initial diagnosis period	Enhances patient and caregiver coping and ability to participate in care planning
**Initial Management**	Educate on chronic illness adjustment and trajectory	Provide anticipatory guidance on psychosocial, cognitive, and developmental concerns	Supports realistic expectations and long-term resilience
	Coordinate interdisciplinary referrals	Connect families with early intervention, mental health services, and respite care	Ensures wraparound care that meets clinical and social needs
	Assist in emergency preparedness and care planning	Clarify home and school emergency protocols; support plan communication	Improves patient and caregiver confidence and reduces hospital-related anxiety
	Address barriers to treatment recommendations	Identify behavioral, emotional, and/or financial obstacles to diet or medication routines	Enhances patient and caregiver ability to sustain treatment and overall metabolic control
**Ongoing Care**	Advocate for work and school accommodations	Help obtain FMLA, assist with employer communication and school IEP/504 planning	Maintains educational engagement and family financial stability
	Clarify and support informed decision-making	Facilitate understanding of treatment options, risks, and life planning	Promotes informed, values-based decisions and family empowerment
	Monitor psychosocial well-being over time	Conduct periodic screenings for stress, depression, and anxiety in patients and caregivers	Supports early intervention for mental health issues and maintains care engagement
	Assist with insurance renewals and appeals	Help navigate benefits recertification and appeal denials for medical food or devices	Prevents treatment interruptions due to administrative gaps
	Provide support for life transitions (eg, adolescence to adult care)	Prepare family and patient for aging out of pediatric systems	Improves continuity of care and fosters patient independence

FMLA, Family and Medical Leave Act; IEP, Individualized Education Program; WIC, Special Supplemental Nutrition Program for Women, Infants, and Children.

With earlier diagnosis through newborn screening and genetic testing and longer life expectancy made possible by medical advances, more individuals with IEMs are living into adolescence and adulthood, creating a need for personalized, multidisciplinary support that adapts to their evolving medical and psychosocial needs over time [2], [6]. Although improvements in the diagnosis and management of IEMs have enhanced prognosis, patients with IEMs remain at significantly higher risk of mortality than age-matched peers, necessitating tailored lifelong support starting at diagnosis [8].

Patients with IEMs and their families often experience challenges related to physical and psychosocial well-being that can impose a significant burden on daily living. Cognitive impairments associated with IEMs can hinder health literacy and lead to compromised metabolic control [9], [10]. Furthermore, treatments often require challenging and intensive lifelong dietary management that entails costly specialized medical foods and supplements, ongoing medical management, and interventions during intercurrent illness (“sick day plans”) [11]. Although curative options such as liver transplant and investigational therapies such as mRNA and gene therapies offer hope, they also introduce new emotional, financial, and social strains—ranging from anxiety, depression, and survival guilt to concerns about equitable and ongoing treatment access [12], [13], [14], [15], [16]. Additional potential stressors include frequent medical monitoring; education challenges (eg, lack of formal education or limited health literacy); food, housing, and transportation insecurities; concerns about care access related to immigration status and/or cultural beliefs; and financial strain related to disease management regimens [17], [18], [19].

Access to public benefits that support the needs of patients with IEMs, often secured by social workers with expertise in IEM management, is highly variable across the US. Eligibility for critical services such as nutritional support, care coordination, and transportation assistance may depend on a complex set of factors, including diagnostic coding, state-specific insurance coverage, and proximity to specialty care centers [20], [21], [22], [23]. While some states offer robust Medicaid waivers or state-funded programs to cover medically necessary foods and supportive services, others provide limited or no coverage [21]. This variability creates disparities in care and can place additional burdens on families, particularly those with limited financial or social resources. As a result, patients with IEMs may experience inconsistent access to essential supports that are crucial for maintaining sustained treatment, quality of life, and long-term health outcomes. In these cases, social workers are well positioned to provide tailored education, offer psychosocial support that considers each patient's developmental stage, and utilize cognitive training to address key physical and psychosocial challenges that could limit overall outcomes.

To effectively care for patients with IEMs, a multidisciplinary holistic approach that incorporates their social and environmental contexts is essential [24]. We argue that social workers must be considered integral members of IEM care teams to help families navigate complex systems, manage psychosocial stressors, and access necessary support, particularly as patients face lifelong challenges that extend well beyond the needs addressed in clinic. This review advocates for the inclusion of social workers in multidisciplinary metabolic care by highlighting the breadth of skills and positive impact social workers offer to patients and families navigating complex medical conditions such as IEMs.

## 2 Impact of social worker integration on enhancing multidimensional health outcomes

**Fig. 1:**
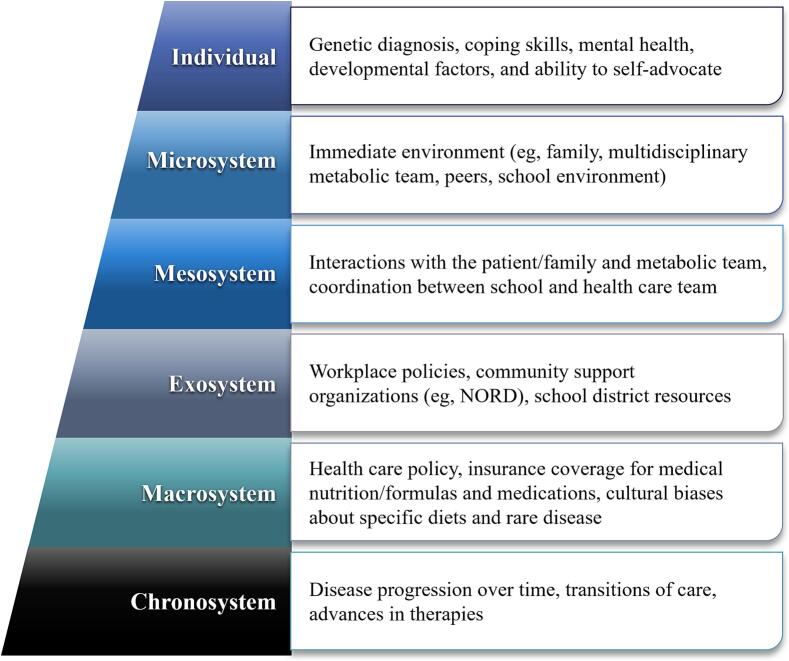
Bioecological Models for Patients With Inborn Errors of Metabolism. IEM, inborn error of metabolism; NORD, National Organization for Rare Diseases. Fig. 1 The care and development of individuals with IEMs are shaped by interacting systems, from the patient's immediate family and clinical team (microsystem) to broader policy and cultural factors (macrosystem), and over time (chronosystem). Family routines, school accommodations, and dynamic relationships with health care providers are central to optimizing adaptation, treatment maintenance, and quality of life for patients and families managing IEMs [75].

Social work is a practice-based profession and an academic discipline that focuses on promoting social change, development, cohesion, and the empowerment of populations facing challenges across a spectrum of domains of daily life. At its core, social work recognizes that a wide range of interconnected factors serve either as opportunities or barriers to well-being and development (eg, historical, socioeconomic, cultural, spatial, political, personal). Due to their unique background and training, social workers have had an essential role in health care since the early 20th century, operating in various settings, including primary and acute care; specialty care; and long-term, palliative, and end-of-life care [25]. A key principle that frames the training of social workers in health care contexts is the holistic biopsychosocial model, introduced by George Engle in 1977, which proposes that health and well-being are impacted by common biological factors in interaction with psychosocial, spiritual, and environmental influences across system levels (Fig. 1) [26], [27].

Accordingly, social workers are increasingly recognized as essential members of multidisciplinary health care teams, contributing psychosocial expertise that complements medical management, particularly in complex and chronic conditions (Table 1). Across various patient populations, integration of social workers into care teams is associated with improved overall health outcomes of patients and reduced health care costs. Specifically, integration of social workers into health care teams has demonstrated benefits such as sustained treatment, enhanced quality of life, and reduced health care utilization [28], [29]. In hospital settings, social workers contribute to significantly lower 30-day readmission rates, especially among older adults and those with complex care needs [30]. In the setting of cystic fibrosis (CF) care, social worker interventions that enhance social support have been associated with fewer reported digestive symptoms and eating disturbances in adults. Social worker integration also reduces rates of depression and anxiety in patients through greater screening and care coordination, which can promote health behavior change and maintenance [31]. In oncology and geriatrics, social workers increase rates of advance care planning and alignment of end-of-life care with patient preferences and are associated with clinically meaningful improvements in overall quality of life [32], [33].

Although guidelines for the care of CF, pediatric cancer, and rare diseases highlight the need for integrated psychosocial screening and support [34], [35], [36], [37], adequate social work staffing across all clinic settings remains a challenge [38]. The US Health Resources and Services Administration predicted a shortfall of more than 10,000 full-time employees in various social work–related professions for 2025 [39]. The US Bureau of Labor Statistics predicted a deficit of 74,000 social workers each year for the next decade, further highlighting the unmet need for patients, families, and health care systems in the United States [39].

Additional studies are needed to properly quantify the multidimensional contributions of social workers in achieving holistic, equitable, and outcome-driven care across rare and complex disease contexts [28]. Future research should prioritize prospective, outcomes-based evaluations that capture the impact of social work on clinical, psychological, and health system metrics across diverse disease contexts. Standardized role definitions and integration models should also be developed within specific treatment areas to guide consistent implementation across care settings [40]. Workforce analyses are also warranted to assess current staffing gaps, inform funding models, and ensure that social workers are adequately resourced to fulfill their expanding scope of responsibilities.

## 3 Integrating social workers into multidisciplinary metabolic care

Though research on the direct impact of social workers in the metabolic genetics field is limited, patients with IEMs and their families experience a disease burden similar to that of other serious chronic conditions, including risk for substantial psychosocial and medical challenges [24]. The impact of a patient's diagnosis and their ability to follow their management regimen is shaped by intrinsic and extrinsic factors, including their ability to self-advocate; their interactions and ability to coordinate with their family, care teams, and school systems; cultural beliefs; and progression of their disease over time. Intrinsic and extrinsic factors also include health and environmental literacy, access to insurance coverage for medical foods and medications, transportation services, school and learning accommodations required for the specific diagnosis, legal support, connection with rare disease resources, and support for social and emotional needs related to chronic disease management. As important members of the metabolic genetics clinic team, social workers perform far-ranging duties that can include assessing the unique constellation of these needs for each patient and family to enable stable and ongoing medical care (Table 1). In addition, social workers are skilled at identifying both local and national resources that may benefit their patient population as these resources vary greatly by region. Social workers must also stay abreast of ongoing changes to resource availability and often advocate at local, regional, and national levels to create or change policies that can improve patient outcomes. Ideally, all individuals with genetic conditions and their families would have access to a specialized, compassionate social worker to help them navigate their care [41], [42]. In care teams without a social worker, these duties fall on health care providers, who may not have the capacity or training to support patients and families in these ways.

The impact of social work in IEMs begins as early as newborn screening, when a positive result ideally triggers medical, psychological, social, and economic support offerings, which are critical components of optimal management and need to be coordinated diligently [24]. Further, IEM management approaches historically involve dietary restrictions to decrease production of toxic metabolites, supplementation of deficient end products, and medications to support residual enzyme function or remove harmful substances [4], [21]. Patients, particularly infants and young children, on these complex treatment regimens typically require frequent laboratory monitoring and medical follow-up, often with multiple providers.

### 3.1 Identifying and addressing social determinants of health (SDOH) as barriers to care

SDOH are nonmedical factors affecting overall health and well-being, health care utilization, and health outcomes [43]. SDOH are largely grouped into 5 domains: economic stability, physical environment, education access and quality, community and social support, and access to health care. These domains encompass a wide spectrum of factors that can directly or indirectly impact patients with IEMs, such as housing, transportation, employment, education level, food insecurity, discrimination, facets of the built environment, social integration or isolation, and language and literacy skills [43], [44]. Recently, greater awareness has resulted in greater prominence of the role of social workers in addressing SDOH as an important and intricately connected component in the care of patients with IEMs, from diagnosis onward [17], [18], [45].

**Fig. 2:**
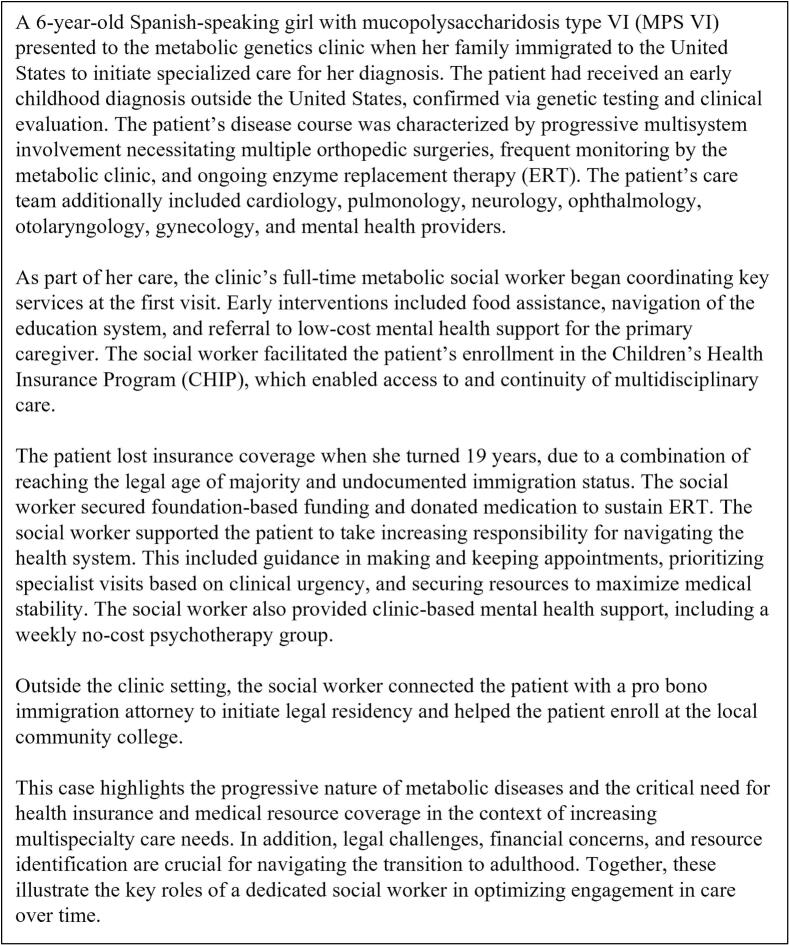
Case vignette: multidisciplinary management of a young adult with mucopolysaccharidosis type VI and complex immigration, legal, and social support needs.

Socioeconomic status and economic factors that lead to housing instability, lack of reliable transportation, and food insecurity will negatively impact a family's ability to effectively implement IEM treatment regimens [46]. Rare disease treatments and medications can be costly; although resources exist, awareness and utilization of financial resources may be low [2], [20]. Education level, language barriers, and limited health literacy may further impede understanding of disease processes and management, resource navigation and utilization, and a patient or family's ability to understand and advocate for appropriate medical care (Fig. 2) [43], [46].

Food insecurity has been reported as a potent factor in the ability of female patients with phenylketonuria (PKU) to follow dietary recommendations [47], highlighting the need to identify and address SDOH barriers within the metabolic clinic. Skilled assessment is needed, as patients and families may not proactively disclose information related to SDOH barriers for fear of how this sensitive information could be perceived and how it may impact medical care [48]. Additionally, patients who experience SDOH-related barriers to care may not seek or accept assistance [49]. Connelley et al. (2025) explored the discrepancy between patients reporting SDOH barriers and those accepting assistance. The authors found that individuals experiencing food insecurity commonly declined assistance because they believed the resources would be unhelpful or redundant or because they felt others might need limited resources more. Patients also declined services because of negative experiences with receiving food assistance, such as local pantries having poor quality food or facing difficulties when attempting to redeem farmers' market vouchers [48]. These challenges may be further compounded in patients with IEMs, for whom dietary management is critical for overall outcomes and whose special diets may necessitate the use of modified low-protein foods and formulas that are often expensive and only readily available from specialty suppliers [11]. The Medical Foods and Formulas Access Act of 2025, federal legislation introduced and currently in the first stage of legislative process, is an example of a resource focused on access to specialized formulas and low protein foods [50]. In addition, organizations such as National Organization of Rare Disorders (NORD) and The Everyday Life Foundation provide key advocacy, education, and additional resources when key gaps are identified. NORD provides a “state report card” that identifies specific state level resources, including local laws and state agency programs. The Everyday Life Foundation provides an opportunity, through a program called “Rare Across America,” to meet and educate local members of Congress to educate them on the issues that are most important to the local community, such as food insecurity and access to low protein foods.

### 3.2 Addressing mental health and Behavioral concerns and promoting appropriate coping strategies

**Fig. 3:**
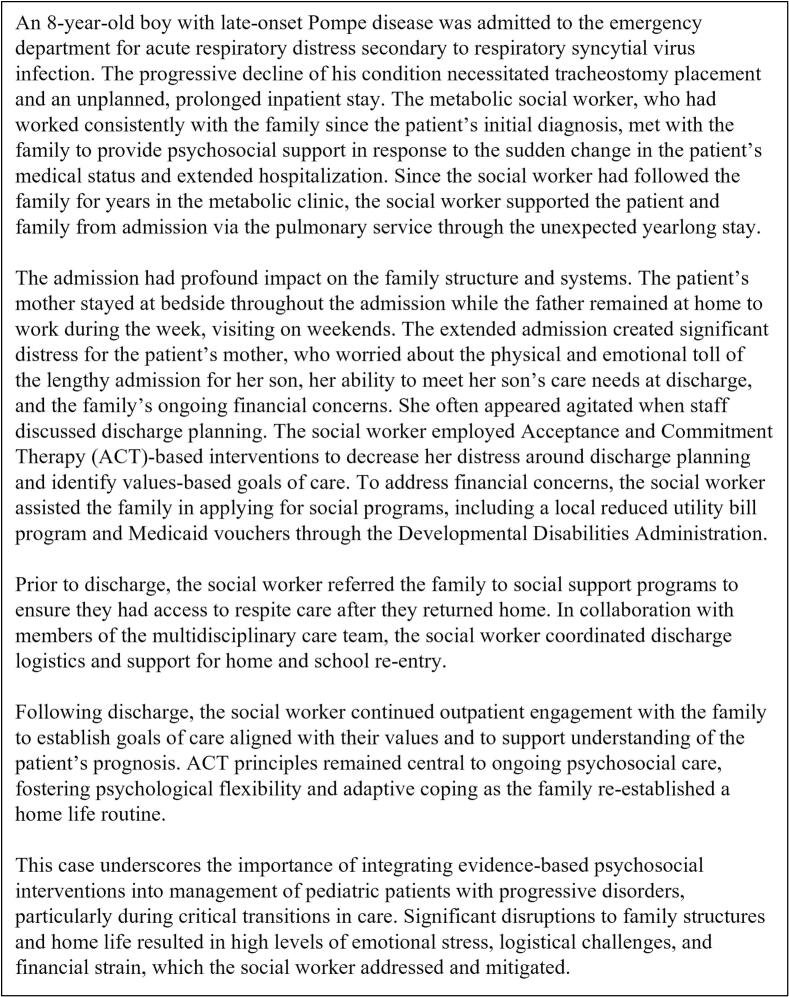
Case vignette: psychosocial support and ACT-based interventions in an 8-year-old with late-onset Pompe disease. Examining the Role of Social Workers Within Clinical Metabolic Genetics Care.

Throughout the diagnostic odyssey, patients and families may experience ambiguity and uncertainty, which evolve over time and can be further amplified by a formal diagnosis [51]. A chronic disease diagnosis not only impacts the individual but also caregivers and other family members [19], [52]. A new diagnosis can elicit feelings of shock, denial, loss, and grief while patients and their families adjust to symptoms of the condition, stress of management, loss of control, financial concerns, and changes in family structure to accommodate the requirements of the disease [19], [52], [53], [54]. Parents of a child with an IEM may struggle with feelings of guilt or blame themselves for the child's condition [50]. Caregivers may experience depression, anxiety, grief or anticipatory loss, and compassion fatigue [50], [55]. In a study of 133 family members of patients across a wide range of chronic conditions, Golics et al. (2013) found that of 92% of participants who reported they were emotionally impacted by their loved one's illness, 20% found it difficult to talk to someone about their feelings. This led to isolation and difficulty coping [56]. Social workers can also support families during acute emergencies and prolonged hospitalizations, which can be a source of significant stress (Fig. 3). By applying crisis intervention models, social workers can effectively counsel and empower patients and families confronting the complexities of a new diagnosis or living with a chronic disease [57]. Licensed clinical social workers may have the ability to seek reimbursement for counseling services provided in both inpatient and outpatient settings, but the ability to bill for services may vary by institution. There is also the need to collaborate across mental health disciplines to address the array of psychosocial and supportive counseling needs of patients, families, and communities living with metabolic disease.

In addition to expected distress over living with a chronic disease, mental health and behavioral disorders are not uncommon for patients with IEMs due to the accumulation of toxic metabolites that disrupt neurodevelopmental processes or neurotransmitter systems. This may lead to emergencies such as delirium or psychosis, as well as personality changes, intellectual disability or developmental delay. Social workers are trained as behavioral health experts to screen for behavioral/mental health problems and refer to neuropsychologists for cognitive testing. The results of these tests are important not only to guide medical care decisions, but also to determine the need for support from community agencies or other adaptive measures that should be taken to help the patient and caregivers succeed with their medical management [58].

In addition to providing mental health and behavioral support to foster coping amid the emotional challenges of disease management, a key role of the metabolic social worker is to support the patient and/or family in developing the management skills needed to successfully address the patient's diagnosis [24]. In the metabolic clinic, this could include teaching families and patients, as they become developmentally or medically able, social workers coordinate with metabolic dietitians on helping patients/caregivers order specialty foods or formula, helping families obtain necessary biochemical testing, and evaluating opportunities to alter the home food environment for successful dietary intervention (eg, establishing more-structured mealtime routines).

### 3.3 Advocating for patients with IEMs at home, school, and work

With advances in diagnostics and medical care, patients with IEMs are living longer and fuller lives but may not be aware of, or have access to, resources that are available to support them in overcoming physical, educational, and employment challenges. Parents and caregivers, in addition to patients themselves, may benefit from support in seeking work situations that can accommodate their caregiver duties and/or the unanticipated hospitalizations that arise for patients with IEMs [59]. Social workers can educate patients and families on available accommodations and help guide families in securing appropriate services for their children through early-intervention, Individualized Education Programs, or 504 plans [60], [61]. Although some adults with IEMs live well independently, others may need support with activities of daily living. In these cases, social workers can guide families through processes related to applying for civil disability benefits, long-term guardianship, medical proxies, and more [59], [62].

### 3.4 Supporting lifelong patient education and treatment maintenance

In the metabolic clinic, patients and families are given the daunting task of understanding complex information for serious and potentially life-threatening disorders. A new diagnosis can make the first clinic visit particularly overwhelming and families are often unable to grasp all the information surrounding a new diagnosis in a single clinic appointment, necessitating ongoing education [63]. Families must process complex medical information, grasp the seriousness of their child's condition, and quickly adopt lifestyle changes, including the addition of new medications and supplements and dietary changes [51], [63]. Social workers can support the dissemination of information to families in an accessible way (ie, addressing all types of learners and literacy levels) and with consideration for each person's specific social, familial, and cultural perspectives. They can also ensure the information is relayed in a manner that is sensitive to the educational needs of the family [57], [64]. Approximately half of all adults have limited health care understanding and struggle to comprehend health information [65]. This is particularly concerning in the metabolic clinic due to the complexity of conditions and severe consequences of not implementing management effectively. A 2016 meta-analysis found that patients with higher levels of health literacy were significantly more likely to correctly take medication and complete other therapies. Interventions supporting health information comprehension were also found to be significantly effective in improving medical management, particularly in vulnerable populations, including low-income and non-White patients [66].

Social workers in specialty clinics often augment support systems for the family during key junctures in care, such as during family planning [57], [64]. Additionally, social workers can help patients and their families reduce psychological distress during the transition from pediatric to adult services by addressing educational, financial, and emotional readiness [67]. Many patients with IEMs maintain lifelong care within the pediatric health care system, which may lead to a lack of emphasis on developing the self-management skills needed to independently care for themselves in adulthood [68]. Along with other members of the care team, social workers can help patients develop the skills needed to care for their disorder more independently, such as scheduling appointments or refilling medications at their pharmacy. Additionally, social workers support families in discussions about appropriate placement of patients who require high levels of care and offer assistance to families as they adjust to a new reality for their loved one [67].

## 4 Advocating for the inclusion of social workers in metabolic care

Social workers can improve patient engagement in health practices, particularly when the social worker is embedded within the metabolic team and introduced to the patient and caregiver(s) early in the care process to build trust and familiarity [24]. Further, by routinely screening for and addressing SDOH concerns, social workers can positively influence a patient's metabolic control. A retrospective review of 255 patients with PKU at a well-resourced metabolic clinic investigated whether social vulnerability, a measure of a community's risk, based on factors such as poverty, housing, and access to transportation (quantified using the Social Vulnerability Index [SVI]), was associated with poor disease control, as measured by blood phenylalanine (Phe) levels. The authors hypothesized that greater social vulnerability would correlate with higher Phe levels due to increased barriers to care. Surprisingly, no significant difference in Phe levels was found between patients from low and high SVI groups. The authors proposed that this finding may reflect the clinic's robust social work and patient assistance services, which helped patients navigate non-medical barriers and sustain complex treatment regimens. This metabolic clinic had a dedicated social worker and engaged a range of social support programs, such as the National Organization for Rare Disorders patient assistance programs for access to low-protein foods, transportation, and medication co-pay assistance, as well as resources from the state that covered lifelong access to essential medical foods. Although further study is needed, these findings suggest a potential beneficial effect of social support programs on metabolic control in patients with IEMs [69].

The evolving genetics workforce has been an area of increasing focus among clinicians and in the peer-reviewed literature as concern for the geneticist shortage grows and the community discusses opportunities to broaden the care of patients with IEMs [6], [70], [71]. In their review of the metabolic workforce, Simpson et al. (2024) discuss opportunities to support the changing landscape, including employing adult-focused ancillary staff, specifically indicating dedicated social workers, to support increasingly common challenges in metabolic care, such as navigating higher education, employment, substance abuse, and socioeconomic challenges [6]. In a case study of a patient with poorly controlled PKU and significant SDOH barriers (limited communication and access to transportation, housing, food, and health insurance), Andrews and McMinimee (2024) suggested that earlier intervention by a metabolic social worker might have improved communication with the family to better address their socioenvironmental barriers to PKU care. They also noted that other clinicians did not have the capacity or expertise to help the family appropriately navigate these challenges [17]. This stands in contrast to a recent case series demonstrating improved Phe levels in 3 patients with PKU for whom a social worker was assigned to address barriers related to housing, medical food attainment, and weekly bloodspot monitoring [24]. These reports further establish the need for integrated, dedicated social worker support within metabolic care.

When social workers are embedded from diagnosis onward, social workers build trust with patients and families and enable early identification and mitigation of any SDOH-related barriers before they escalate into more-serious problems [24]. Recognition of the value of social workers within genetics services has a longstanding history. In 2003, the National Association of Social Workers published standards to expand training in key genetics competencies and ensure social workers have the tailored skills needed to work effectively with families receiving genetics care. These standards focus on 4 core areas: (1) promoting equitable access to genetic testing and services, (2) providing foundational genetics knowledge to support social workers' understanding of inherited disease patterns, (3) encouraging self-reflection on personal values and beliefs that may influence counseling on sensitive topics such as reproduction, medical intervention, and disabilities; and (4) fostering cultural competency [64]. Contrastingly, while specialized training in IEMs is recommended for social workers treating this population so they can be aware of the unique burdens on these patients and families [59], formal educational opportunities for metabolic social workers do not currently exist, with most learning occurring through on-the-job training and experience. Formalized instruction will need to be created, funded, and implemented by experts in metabolic social work. Until that time, current metabolic training organizations, such as the North American Metabolic Academy, sponsored by the Society for Inherited Metabolic Disorders, and Metabolic University, sponsored by Genetic Metabolic Dietitians International [6], could consider integrating social workers into their programming. Two emerging organizations focused on addressing the unique psychosocial needs of patients and families include Social Work in Genetics Collaborative, established in 2025, and the National PKU Alliance Psychosocial Committee.

Encouragingly, social work is one of the fastest growing professions in the United States, and the US Bureau of Labor Statistics estimates a 7% increase in health care social workers by 2033 [72]. This high level of growth offers insight into how metabolic clinics can continue to evolve to provide comprehensive and patient-centered care that addresses psychosocial well-being. Metabolic clinics should advocate for dedicated social work support to meet the growing needs of patients with IEMs. A key barrier to the inclusion of social workers in metabolic clinics is the concern regarding low reimbursement potential. In recent years, an increased focus on improving access to mental health services led the Centers for Medicare and Medicaid Services to enact changes that allow for improved billing for services that address mental health support and health-related social needs, including health behavior and SDOH risk assessments, along with intervention services [73]. Due to continuous updates in this area, metabolic clinics should work to identify optimal billing strategies to support appropriate resourcing and ensure productivity of social workers is captured. Funding opportunities for social workers may also be available through grant-funded initiatives, such as state newborn screening programs.

## 5 Summary and conclusions

The integration of social workers into metabolic clinics would address critical gaps in the care of patients with IEMs to ensure optimal patient care from multidisciplinary health care teams. Social workers are uniquely equipped to lead sensitive SDOH screening, identify corresponding patient/caregiver needs, and address unique IEM-related barriers [74]. This critical addition to the structure of the metabolic clinic supports the evolution of metabolic care as the number of patients with rare disease grows. Three key concepts emerged from this review and discussion: (1) patients with IEMs and their families face significant psychosocial burdens that impact their ability to meet management goals; (2) social workers are the ideal clinicians to assess and address barriers to care and improve outcomes for patients with IEMs; and (3) social workers in metabolic genetics should be fully embedded within the multidisciplinary team, be knowledgeable of the intricacies of IEM care, and be introduced to families at an early stage in disease management.

Social work is a rapidly growing field, which provides an opportunity for metabolic clinics to engage this discipline to ensure that psychosocial needs are being addressed and barriers to care reduced for patients with IEMs. While the integration of social workers into metabolic care is gaining interest, it is still relatively new and most clinics do not currently employ a dedicated social worker. We strongly recommend that social workers be prioritized as key members of the multidisciplinary metabolic team. Clinics can look to other disease states, such as CF and cancer, for insights on optimal staffing ratios and opportunities to capture metabolic clinic revenue. Clinics that do not currently have adequate social work support must be diligent in ensuring the psychosocial needs of patients are not overlooked by implementing screening protocols for SDOH and mental health barriers, collating and maintaining an updated list of resources to act as a first line of support when SDOH or mental health concerns are identified, and developing a referral network for key patient concerns.

## Credit authorship contribution statement

**Soo Shim:** Writing – review & editing, Writing – original draft, Investigation, Conceptualization. **Diana Cory:** Writing – review & editing, Writing – original draft, Investigation, Conceptualization. **Gailon Wixson:** Writing – review & editing, Writing – original draft, Investigation, Conceptualization. **Abigail Hata:** Writing – review & editing, Writing – original draft, Investigation, Conceptualization. **Allison Werner-Lin:** Writing – review & editing, Writing – original draft, Investigation, Conceptualization.

## Statements and declarations

Funding for writing and editorial support, author coordination, manuscript submission, and open access was provided by Amgen Inc. The authors confirm independence from the sponsor; the content of the article has not been influenced by the sponsor. No author received compensation for the preparation of this manuscript.

## Funding sources

Funding for writing and editorial support, author coordination, manuscript submission, and open access was provided by Amgen Inc.

## Declaration of competing interest

S. Shim and G. Wixson have received consulting fees from Amgen Inc. for advisory and consulting activities. The other coauthors have nothing to declare.

## Data Availability

No data was used for the research described in the article.
